# Novel ELISA method as exploratory tool to assess immunity induced by radiated attenuated sporozoites to decipher protective immunity

**DOI:** 10.1186/s12936-017-2129-9

**Published:** 2017-11-29

**Authors:** Trey A. Knepper, Elizabeth H. Duncan, Tatyana Savransky, Elke S. Bergmann-Leitner

**Affiliations:** 1 Malaria Vaccine Branch, U.S. Military Malaria Research Program, 503 Robert Grant Ave, 3W53, Silver Spring, MD 20910 USA; 2 Division of Entomology, Walter Reed Army Institute of Research Walter Reed Army Institute of Research, 503 Robert Grant Ave, 3W53, Silver Spring, MD 20910 USA

**Keywords:** Malaria, Immunity, Correlates of protection, Plasmodium, Assay development, Sporozoite, Antibodies

## Abstract

**Background:**

Whole parasite vaccines provide a unique opportunity for dissecting immune mechanisms and identify antigens that are targeted by immune responses which have the potential to mediate sterile protection against malaria infections. The radiation attenuated sporozoite (PfSPZ) vaccine has been considered the gold standard for malaria vaccines because of its unparalleled efficacy. The immunogenicity of this and other vaccines continues to be evaluated by using recombinant proteins or peptides of known sporozoite antigens. This approach, however, has significant limitations by relying solely on a limited number of known pathogen-associated immune epitopes. Using the full range of antigens expressed by the sporozoite will enable the comprehensive immune-profiling of humoral immune responses induced by whole parasite vaccines. To address this challenge, a novel ELISA based on sporozoites was developed.

**Results:**

The SPZ-ELISA method described in this report can be performed with either freshly dissected sporozoites or with cryopreserved sporozoite lysates. The use of a fixative for reproducible coating is not required. The SPZ-ELISA was first validated using monoclonal antibodies specific for CSP and TRAP and then used for the characterization of immune sera from radiation attenuated sporozoite vaccinees.

**Conclusion:**

Applying this simple and highly reproducible approach to assess immune responses induced by malaria vaccines, both recombinant and whole parasite vaccines, (1) will help in the evaluation of immune responses induced by antigenically complex malaria vaccines such as the irradiated SPZ-vaccine, (2) will facilitate and accelerate the identification of immune correlates of protection, and (3) can also be a valuable assessment tool for antigen discovery as well as down-selection of vaccine formulations and, thereby, guide vaccine design.

**Electronic supplementary material:**

The online version of this article (10.1186/s12936-017-2129-9) contains supplementary material, which is available to authorized users.

## Background

To date, only whole parasite vaccines have been able to reproducibly induce high vaccine efficacy against malaria infection in humans [immunization under chemoprophylaxis (CPS), immunization with radiation attenuated sporozoites (PfSPZ), immunization with genetically attenuated sporozoites (GAS)] (reviewed in [1]). The question which SPZ-derived antigens mediate this protection has been the focus of intensive investigations, and the analysis of immune responses of vaccinees, who received whole parasite vaccines, has revealed novel antigens, such as the CelTOS protein, as well as some other antigens that are currently being evaluated for their ability to induce protective immunity when used in recombinant vaccines.

Major efforts have been undertaken to make the radiation attenuated sporozoite vaccine (PfSPZ) a viable alternative to recombinant vaccines against malaria. The PfSPZ-vaccine is based on the observation that large numbers of infectious bites from irradiated mosquitoes mediate sterile protection. The original approach required the immunization to take place close to a large insectary capable of supplying sufficient numbers of radiation attenuated, infected mosquitoes [2, 3]. More recently, radiation attenuated sporozoites have been cryopreserved and are eventually delivered by needle and syringe [4]. Initial studies with this vaccine demonstrated that needle-based immunizations targeting the skin or muscle only had limited vaccine efficacy [5]. This raises two questions: (1) what is the difference between vaccination with irradiated mosquitoes and injection of purified irradiated sporozoites in regards to immunomodulatory saliva components; (2) what is the impact of the delivery route and/or sporozoites dose of the needle-based vaccination (reviewed in [2]). Several clinical trials are underway or planned to address these questions and to establish a vaccine regimen that will provide complete, sterile and long-lasting protection. While the only reliable readout for vaccine efficacy currently available is a controlled human malaria infection (CHMI), the ability to identify a correlate or a surrogate marker of protection would greatly reduce the need for CHMIs thereby reducing the costs associated with vaccine design and accelerate the time line for an efficacious vaccine.

Apart from the obvious desire to develop a protective malaria vaccine, whole parasite vaccines also offer the opportunity to study immune mechanisms that lead to sterile protection. These findings can then be used to develop recombinant vaccines that emulate a sporozoite-based vaccine. Early studies with the radiation attenuated sporozoite (RAS) vaccine in preclinical models identified CD8^+^ T cells, IFN-γ, and antibodies as being essential for providing protection [6]. While the role of CD8^+^ T cells in mediating protection in human vaccinees is less supported by data [7], they have been shown to be crucial in preclinical models [6]. This could, in part, be due to the fact, that these cells may not be present in sufficiently large numbers in human peripheral blood, thus eluding detection. In contrast, the important role of antibodies in mediating protection induced by a variety of vaccine platforms has been well demonstrated [7–9].

Antibodies induced by whole parasite vaccines are mostly measured using conventional ELISA methods that employ either the full-length circumsporozoite protein (CSP) or the central repeat region of the CSP as plate antigen. While CSP is the major surface protein, using only a single antigen to assess the immunogenicity and magnitude of the vaccine induced humoral immune response provides quite limited insight into the vaccine-induced humoral immune response and may result in flawed conclusions and misdirect the search for immune correlates of protection.

This report describes a novel, simple, and highly reproducible ELISA protocol (SPZ-ELISA) based on employing *Plasmodium falciparum* sporozoites as plate antigen. To date, anti-sporozoite serological responses are captured by performing immunofluorescence assays with sporozoites [10–12]. Evaluating immune responses by microscopy is labour intense and, unless imaging systems are available, quantitation may be limited and standardization between laboratories is challenging. The high-throughput and ease of ELISA based assays offer an opportunity to evaluate serological responses recognizing sporozoites. There have been two reports on genetically attenuated *Plasmodium yoelii* sporozoite vaccines where sporozoite lysates were used in an ELISA format [13, 14]. To date, there are no reports or protocols describing the use of *P. falciparum* sporozoites as plate antigen in an ELISA. In reports where researchers utilize ELISA assays to detect or measure the number of sporozoites, monoclonal antibodies specific for CSP are used to capture sporozoites in a sandwich ELISA format [15] and such an assay has not been validated for the assessment of vaccine specific responses. The assay was validated using well-established monoclonal antibodies to CSP and Thrombospondin related adhesive protein (TRAP), and applied to pooled sera to establish the usefulness of the SPZ-ELISA as a novel tool for comprehensively evaluating ab responses to antigenically complex malaria antigens. It is proposed that this assay format will be able to serve as an additional tool for the ongoing search for immune correlates of protection against malaria.

## Methods

### Sporozoite preparation

Sporozoites were prepared by dissecting mosquitoes 16–20 days post blood feed using the Ozaki method [16]. Sporozoites were either immediately coated onto ELISA plates or frozen as pellets for use as lysates.

### Antibodies

For the detection of sporozoite antigens, the following antibodies were used: 2A10 (anti-CSP, BEI resources NIAID), clone SAI171C-5E2 (anti-TRAP, BEI resources), clones TH1 and TH3 (kind gift of Dr. Ted Hall, WRAIR), de-identified serum pools from radiation attenuated sporozoite-immunized (RAS) vaccinees [17]. The “protected” pool consisted of six subjects and the “non-protected” pool consisted of five subjects. Mouse monoclonal 1D9 (ATCC, Manassas, VA) and pre-immune serum pool from all RAS vaccinees were used to determine background reactivity against sporozoites. Secondary antibodies (goat-anti mouse IgG-AP, goat-anti-human IgG-AP) were purchased from Southern Biotech (Birmingham, AL).

### ELISA

Freshly dissected sporozoites or thawed sporozoite pellets were suspended to the desired concentration with PBS (pH 7.4) and plated at 30 μl/well in Immulon 2HB plates (Thermo Scientific Waltham, MA). Plates were incubated for 2 h at room temperature (RT). The liquid was gently removed from wells and plates were either allow to air dry or fixed by adding 50 μl/well fixative (1% paraformaldehyde, 3% glutaraldehyde, or methanol). Plates were then blocked with PBS + 1% BSA (50 μl/well) for 1 h at RT. Primary antibodies were diluted with PBS + 1% BSA, added to the respective wells (50 μl/well), and plates were incubated for 2 h at RT. Unbound antibodies were removed by washing the plates three times with PBS. Secondary antibodies were diluted with PBS + 1% BSA (1:200) and added to each well (50 μl/well) for 1 h at RT. Plates were then washed with PBS and substrate (BluePhos, SeraCare, Gaithersburg, MD) was added. Plates were read on a SpectraMax M2 plate reader (Molecular Devices, Sunnyvale, CA) at 630 nm absorption.

### Statistical analysis

Statistically significant differences between the various assay conditions were determined by using two-sided T tests (Minitab 17, State College, PA).

## Results and discussion

### Coating conditions

**Fig. 1:**
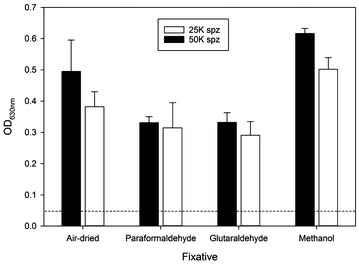
Comparison of coating conditions. *Plasmodium falciparum* sporozoites were coated at 50,000 (black bar) or 25,000 (white bar) sporozoites per well and then either air-dried or fixed using the fixatives indicated on the x-axis. Plates were then probed with a CSP-specific monoclonal antibody (clone 2A10) or control mAb 1D9. Data are expressed as mean OD (± SD) of quadruplicates of a representative experiment. The dashed line indicates the background activity [maximal ELISA signal obtained with the control mAb (OD < 0.05 for all conditions)]

First, the sporozoite concentration for coating was determined for both intact and lysed sporozoites (Additional file 1). When coating with intact sporozoites, as few as 500 sporozoites per well can be detected; the linear range for detection is between 1250 and 5000 sporozoites. Coating the ELISA plates with lysed sporozoites reduced the signal strength compared to intact parasites. The next step in assay development was to determine whether intact sporozoites or sporozoite lysates are more useful as plate antigen. Binding of the sporozoite was measured by probing the plates with an anti-CSP monoclonal antibody (clone 2A10) since CSP is the major surface protein and abundantly expressed. Various fixatives were tested to immobilize sporozoites onto the plastic; these conditions were compared to “air dried” sporozoites. This comparison showed that sporozoites dried onto the surface as well as methanol fixation and resulted in the strongest ELISA signals (Fig. 1). Therefore, subsequent assay optimization was based on comparing these two conditions. Air drying the sporozoites or lysates onto the assay plates is preferable since no alterations of epitopes are introduced, which is a typical consequence of fixatives. However, the use of a fixative may increase the stability of the plates since proteases are inactivated.

### Immune recognition of intact versus lysates by sporozoite specific antibodies

**Fig. 2:**
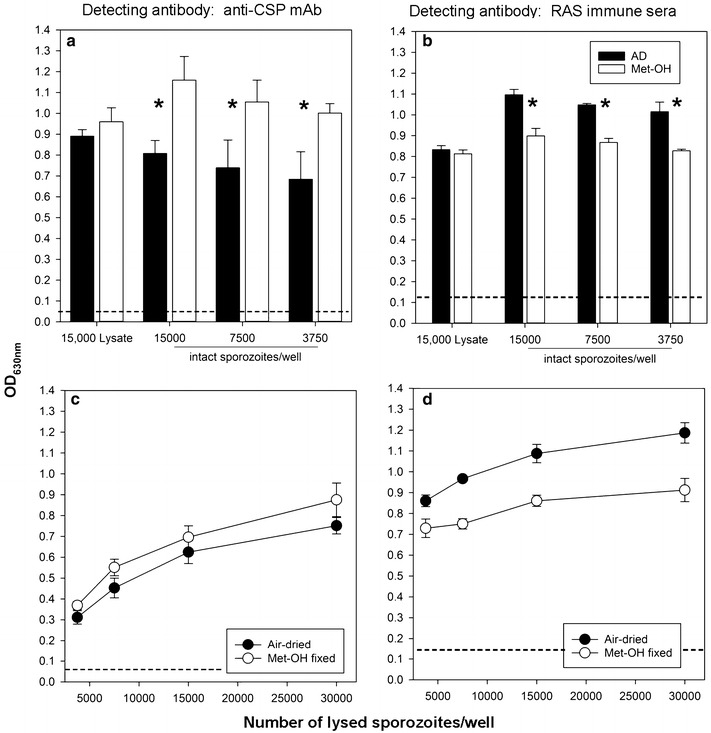
Differential reactivity of antibodies with air-dried vs. methanol fixed sporozoites. Various concentrations of intact sporozoites (**a**, **b**) or sporozoite lysates (**c**, **d**) were plated and air-dried or methanol fixed. Plates were then probed with either a CSP-specific mAb (2A10) (**a**, **c**) or pooled sera from RAS immune subjects (**b**, **d**). The dashed lines indicate the background activity [ELISA signal obtained with either the control mouse mAb 1D9 (OD < 0.05) or pre-immune serum pool (OD < 0.12)]. Asterisks indicate statistical significance (p < 0.05, paired T test)

The coating of ELISA plates with either intact sporozoite or sporozoite lysates was tested to determine which of the sporozoite preparations produces the strongest and most specific signal. Sporozoite or lysate coated plates were probed with either anti-CSP mAb (2A10) (Fig. 2a) or human pooled sera from RAS immunized volunteers (Fig. 2b). The data demonstrate that the CSP-specific mAb 2A10 has higher reactivity with methanol-fixed sporozoites (p = 0.02, paired T test) while RAS vaccine-induced antibodies react significantly better with air dried, intact sporozoites (p < 0.04, paired T test). The same differential reaction pattern is seen when testing sporozoite lysates: 2A10 reacting stronger with methanol fixed lysates, RAS immune antibodies react better to air dried lysates (Fig. 2c, d). The most likely explanation for the different reaction pattern is the fine specificity of the monoclonal vs. the polyclonal antibodies: mAb 2A10 is specific for the central repeat region of CSP, and fixation may not affect the structure of the repeat. Interestingly, fixation seems to increase the accessibility of this epitope as indicated by higher reactivity with the CSP-specific monoclonal antibody. The RAS immune serum pool is expected to react with many different epitopes on different SPZ-antigens, and fixation may impact some of these epitopes resulting in reduced recognition by immune serum. Intact sporozoites as plate antigen present epitopes in their native conformation and density thus resulting in better recognition. Once sporozoite lysate is used to coat ELISA plates, the difference between the air dried and methanol fixation is not as prevalent anymore. Lysates did not produce a stronger ELISA signal compared to intact sporozoites (Fig. 2), which suggests that RAS immune antibodies only recognize surface antigens of the sporozoite and not cytoplasmic antigens. However, it could be that the lack of a difference between whole sporozoite and lysates is due to a dominant CSP response, which will be found in excess in both conditions. This finding was unexpected and warrants further studies, which were outside the scope of the current study. Ideally, any key reagent used in an ELISA should be produced as a larger batch to minimize inter-assay variability. Being able to dissect large numbers of mosquitoes and freezing single assay-aliquots is convenient, and this allows larger set of sera to be assessed under the same conditions. Therefore, the subsequent experiments were performed with lysates only.

### Air dried sporozoite material has higher immune reactivity with RAS immune antibodies

**Fig. 3:**
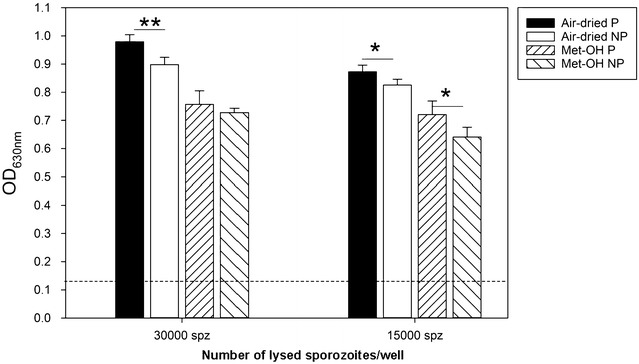
Air-dried sporozoite react significantly better with polyclonal RAS immune antibodies. Lysates were either dried onto ELISA plates or fixed with methanol onto the plate’s surface. Plates were then probed with two serum pools from protected (P) or non-protected (NP) individuals at a 1:2000 dilution. Asterisk indicates statistical significance (paired T test). Data are expressed as mean OD (SD). The background OD with the pre-immune serum pool was 0.13 ± 0.04 (30,000 spz) and 0.12 ± 0.05 (15,000 spz)

Coating ELISA plates by either air drying the sporozoite lysate onto the plastic or fixing the lysate with methanol changes the accessibility of epitopes (Fig. 2). This led to the question whether there is a qualitative or quantitative difference in the reactivity of polyclonal, RAS immune sera depending on the protective status of the vaccinees. Since the goal of this study was the development of a new readout method and not vaccine evaluation, serum pools from RAS immunized subjects that were either protected or not protected after CHMI were generated and tested in the SPZ-ELISA (Fig. 3). Using serum pools also sets a higher bar as the responses of each of the volunteers must be significantly different from the other group to yield a statistically significant difference.

RAS-immune sera reacted significantly better with air dried than with methanol fixed lysates (p < 0.05, paired T test) regardless of the protective status of the vaccinees. When comparing the protected vs. non-protected groups, serum from protected vaccinees generated a significantly stronger signal when using air dried lysates (p < 0.01, paired T test). The reactivity of the protected vs. non-protected serum pools was not as significant when using methanol fixed sporozoite lysates and when coating with the higher antigen concentration; only at the lower SPZ concentration the difference was significant (p = 0.04, paired T test). These results suggest that the sporozoite ELISA may be a valuable tool for the analysis of whole sporozoite vaccines.

### Reactivity of sporozoite-specific monoclonal antibodies in the SPZ-ELISA

**Fig. 4:**
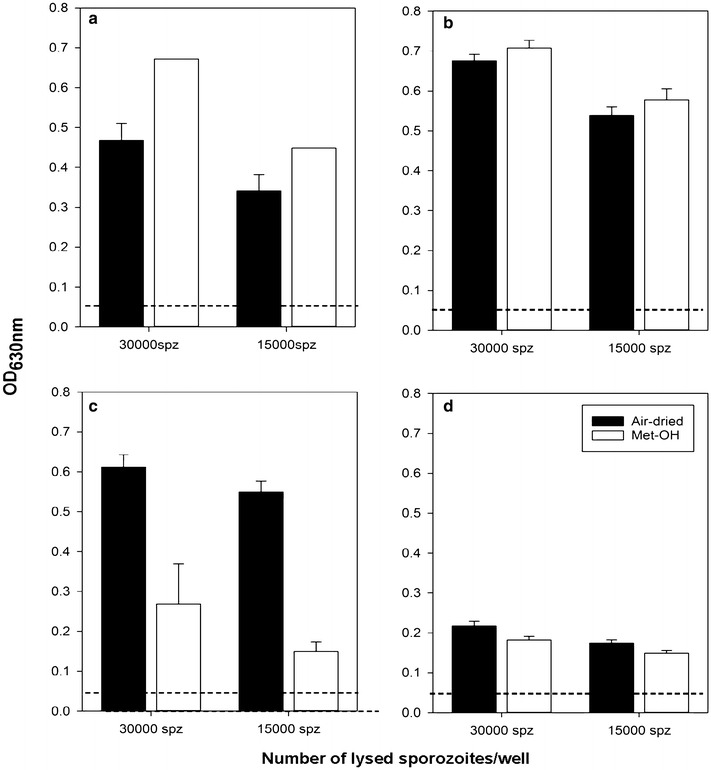
CSP specific antibodies show distinct reaction patterns when testing air dried vs. methanol-fixed sporozoites. **a** CSP repeat-specific mAb 2A10, **b** CSP specific mAb TH3, **c** CSP-C-term specific TH1, **d** TRAP-specific mAb 5E2. The dashed lines indicate the background activity [ELISA signal obtained with the control mouse mAb 1D9 (OD < 0.05)].

Lastly, it was determined whether the SPZ-ELISA can be used to distinguish specificities of various CSP-specific monoclonal antibodies, and whether other sporozoite specific antigens such as TRAP can be detected (Fig. 4). While the CSP repeat-specific mAb 2A10 showed stronger reactivity to methanol-fixed sporozoites, there was a clear distinction between the response of CSP mAbs TH3 and TH1: the epitope for TH3 is believed to be upstream of the C-terminus, while TH1 binds within the Pf16 epitope reported earlier [18, 19]. mAb TH1 recognized air dried sporozoites significantly better which may indicate that methanol-fixation alters or obstructs the C-terminal TH1 epitope. RAS-immune sera reproducibly produced stronger reactivity in the SPZ-ELISA than any of the CSP specific mAbs tested thus indicating that additional epitopes and/or antigens are recognized by these sera. This is not surprising since monoclonal antibodies only recognize a single epitope. To test reactivity with other sporozoite-specific mAbs, the TRAP-specific mAb 5E2 was tested in this assay and showed a reproducible signal above background. Interestingly, with this mAb no significant difference was observed when comparing air dried vs. methanol-fixed sporozoites.

## Conclusion

The present study describes the development of an experimental approach to assess immune responses induced by malaria vaccines, in particular whole parasite vaccines. The use of reagents that can easily be made available to any laboratory was a major consideration and, therefore, the use of sporozoite lysates was tested regarding its impact on the resulting immunoreactivity in the ELISA method. The method described here represents an asset for the evaluation of immune responses induced by antigenically complex malaria vaccines such as the irradiated SPZ-vaccine and will, therefore, facilitate and accelerate the identification of immune correlates of protection.

## Additional file

**Additional file 1.** Sensitivity of sporozoite specific ELISA. Plates were coated with various concentrations of either intact sporozoites (panel A) or sporozoite lysate (panel B). Sporozoites were detected using a CSP-specific mAb (clone 2A10). Data are expressed as mean (SD) of two independent experiments.
