# Supplementary material for: Novel ELISA method as exploratory tool to assess immunity induced by radiated attenuated sporozoites to decipher protective immunity
Source: Malar J. 2017 Nov 29;16:484. doi: 10.1186/s12936-017-2129-9 (PMC5707923; doi:10.1186/s12936-017-2129-9)
Supplement: Supplementary file 1 — Additional file 1. Sensitivity of sporozoite specific ELISA. Plates were coated with various concentrations of either intact sporozoites (panel A) or sporozoite lysate (panel B). Sporozoites were detected using a CSP-specific mAb (clone 2A10). Data are expressed as mean (SD) of two independent experiments. [file 12936_2017_2129_MOESM1_ESM.docx]

**Additional File**

**Novel ELISA method as exploratory tool to assess immunity induced by radiated attenuated sporozoites to decipher protective immunity**

Trey A Knepper, Elizabeth H Duncan, Tatyana Savransky, Elke S Bergmann-Leitner

*Assay sensitivit.* Performing dose titration experiments comparing the two coating conditions (air-drying vs. methanol-fixation). The lower level of detection is approx. 500 sporozoites per well. The use of lysates may be more convenient as large batches of sporozoites could be dissected, prepared, frozen, and aliquots thawed for experiments. Alternatively, cryopreserved, commercially available for laboratories that do not have access to an insectary) sporozoites could be used for this assay.


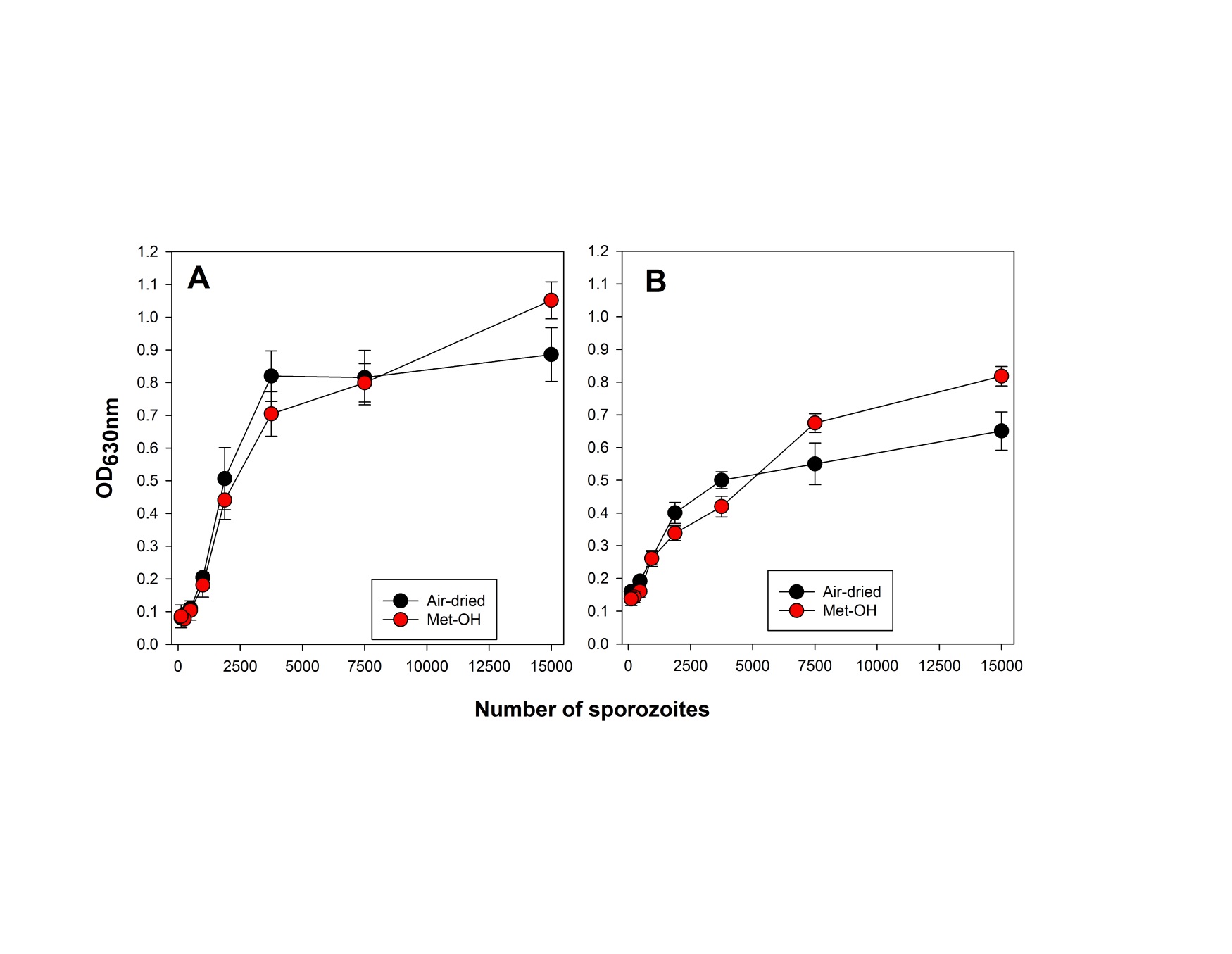


Additional figure 1: Sensitivity of sporozoite specific ELISA. Plates were coated with various concentrations of either intact sporozoites (Panel A) or sporozoite lysate (Panel B). Sporozoites were detected using a CSP-specific mAb (clone 2A10). Data are expressed as mean (SD) of two independent experiments.
